# Moving through adulthood: The lived experience of Irish adults with PKU

**DOI:** 10.3389/fpsyg.2022.983154

**Published:** 2022-09-13

**Authors:** Mary-Ellen O'Shea, Bernadette Sheehan Gilroy, Anna-Marie Greaney, Anita MacDonald

**Affiliations:** ^1^School of Health and Social Sciences, Munster Technological University, Kerry, Ireland; ^2^Birmingham Women's and Children's NHS Foundation Trust, Birmingham, United Kingdom

**Keywords:** phenylketonuria, lived experience, low protein diet, adults, qualitative research, rare condition

## Abstract

**Background:**

This paper represents a portion of the findings from one of the first research studies eliciting the lived experience of adults with an early diagnosis of Phenylketonuria (PKU) living in Ireland. Ireland has one of the highest prevalence rates of PKU in Europe, however, little is known about the experience of Irish adults with PKU. Furthermore, Ireland is one of the first countries in the world to introduce neonatal screening followed by the introduction of long-term dietary therapy over 50 years ago. This study presents the first comprehensive assessment of the lived experience of Irish adults with PKU on long term dietary therapy.

**Methods:**

Narrative data was collected from eleven self-selected participants, using semi-structured interviews. The interviews were divided into five sections focused on eliciting a holistic understanding of the lived experience of adults with PKU living in Ireland. Thematic analysis was guided by Colaizzi's Framework (1978) in conjunction with NVivo qualitative data analysis software.

**Findings:**

Findings from the original research encompassed a broad understanding of the lived experience of adults with PKU living in Ireland, including factors influencing dietary therapy and managing PHE blood levels. The themes being discussed within this article are those which appear to be least represented within current literature: living with PKU, including reproductive health, the importance of self-management and establishing routine, support networks in adulthood and concerns regarding aging with PKU.

**Conclusion:**

It was evident from the findings that a diagnosis of PKU can influence how adults with PKU may experience aging and their own mortality. These findings offer new insight into the vulnerability attached to the experience of aging with PKU and may be beneficial to advocacy groups and for future development of policy and practice.

## Introduction

Phenylketonuria (PKU) is a rare genetic condition that affects the body's ability to metabolize the amino acid phenylalanine (PHE), which is found in many foods (Blau et al., 2010). PKU is characterized by a build-up of PHE in the blood and brain which can potentially result in irreversible intellectual disability, developmental delay and psychiatric symptoms (Van Wegberg et al., 2017). Adherence to lifelong dietary therapy (DT) is recommended within the first 4 weeks of life to prevent neurological damage (Van Wegberg et al., 2017). Despite the success of DT, it is evident from the literature that it is complex and adherence is challenging (Van Wegberg et al., 2017; Ford et al., 2018a).

Ireland was one of the first countries to introduce neonatal screening using the heel prick test in 1966 (Health Service Executive, 2022), and has one of the highest incidences of PKU in Europe with 1 in every 4,500 cases being identified (Loeber, 2007). From a clinical perspective, the introduction of neonatal testing in the 1960s and the development of lifelong DT has had a positive impact on the lives of people with PKU (Bugard et al., 2017). Furthermore, PKU research has had a significant impact in the development of neurosciences, influencing our understanding of human metabolism, metabolic conditions and brain development (Christ, 2003). For example, the development of neonatal testing and introduction of lifelong DT (Bugard et al., 2017) have since been adapted and applied to other metabolic conditions, e.g., Maple Syrup Urine Disease (Paul and Brosco, 2013). However, there is very little known about the lived experience of adults with PKU (AwPKU) living in Ireland. Therefore, the primary aim of the original research study was to elicit the lived experience of adults with an early diagnosis of PKU on lifelong DT living in Ireland.

## Materials and methods

### Recruitment and participant profile

Participants consisted of 11 self-selected adults, male (*n* = 7) and female (*n* = 4), ranging in age from 21 to 49 years of age (see Table 1). Inclusion criteria consisted of adults over the age of 18 years with a diagnosis of PKU from birth, on long term DT living in Ireland at the time of data collection. Recruitment commenced in November 2018 and lasted approximately 1 year. All participants were in full time employment or education. The PKU Association of Ireland (PKUAI) acted as gatekeepers in the recruitment process of participants for the original research study. The PKUAI advertised the research study on their website, social media platforms and discussed it at member meetings.

**Table 1:** Participants profile.

**Gender**	**Age range** **(in years)**	**Total number (n) in adult health services**	**Total number (n) transitioning between services**	**Total number (n) with siblings with PKU**	**Total number (n) with offspring**	**Total number (n) with offspring with PKU**
Female (n = 4)	20yrs – 30yrs (n = 2) 40yrs – 50yrs (n = 2)	3	1	2	1	0
Male (n = 7)	20yrs – 30yrs (n = 1) 31yrs – 40yrs (n = 2) 41yrs – 50yrs (n = 4)	6	1	1	3	0

### Methods

Interviews are considered the most suitable method of data collection within phenomenological research (Padilla-Diaz, 2015). Therefore, phenomenological, semi-structured interviews were used to collect narrative data. An interview guide was developed using themes that emerged from a review of the literature. It was then divided into five sections focused on eliciting a holistic understanding of the lived experience of AwPKU living in Ireland; Living with PKU, Treatment and Monitoring, Relationships, Sexuality and Fertility, Psychosocial Issues and the Future and PKU (see Table 2). A pilot interview (n = 1) was carried out prior to initiating the formal interview process. Data obtained from the pilot interview was excluded from the official data collection and analysis. Due to the limited PKU population of Ireland, the pilot participant resided in Spain but fulfilled all other research inclusion criteria. Following the pilot interview process, some adjustments were made to the interview guide regarding phrasing, placement and timing of questions. Interviews were conducted by the researcher (Mary-Ellen O'Shea) and lasted, on average, 1 h and 30 mins.

**Table 2:** Interview guide.

***Introduction to Living with PKU***
**What is it like to live in Ireland with PKU?**
*Other probing questions as needed:*
Do you see PKU as an illness? Please explain
How do you manage PKU and everyday life?
***PKU Dietary Treatment and Monitoring***
**Tell me about the low protein diet, what is it like?**
*Other probing questions as needed:*
How long does it take to prepare?
Tell me about the availability of medical foods in Ireland.
Cost of adhering to diet, is it sustainable?
Do you ever eat food you probably shouldn't or in secret? How do you feel before and afterwards? How do you manage these feelings?
How often within the last 2 years have you monitored your blood PHE levels?
Are you aware of fluctuating PHE levels without testing? If so, how?
Did you transfer from pediatric to adult service? How did this impact you?
What are the available support services like? Are you aware of what's available?
***Relationships, Sexuality and Fertility***
**What is like to be a Man/Woman with PKU?**
*Other probing questions as needed:*
Tell me about establishing/maintaining relationships in your life
Has having PKU influenced your decision to have children?
If you have children, how were you supported throughout the pregnancy?
***Psychosocial Issues***
**Does PKU impact on your overall wellbeing? How?**
**Do you partake in physical activity/sport/hobbies? Are there any associated benefits or barriers to participation in activities due to PKU?**
Do you travel? How do you manage PKU when traveling?
***The future and PKU***
Are you aware of new developments in the treatment? How will this affect you?
*Other probing questions as needed:*
Are there any changes you would like to see being made to current treatment or management practices of PKU?
**Have you anything else you would like to add?**

### Analysis

Each interview was recorded and transcribed verbatim prior to analysis. Colaizzi (1978) framework was the primary tool of thematic analysis used in conjunction with the computer-assisted qualitative data analysis software, NVivo, where thematic analysis and coding commenced. Significant statements were annotated, and the larger body of narrative data was broken down into codes and categorized for further analysis. The codes were then grouped into themes which ultimately led to the development of overarching themes, and sub themes within. See Table 3 for an overview of all thematic areas with those reported on in this paper being highlighted.

**Table 3:**
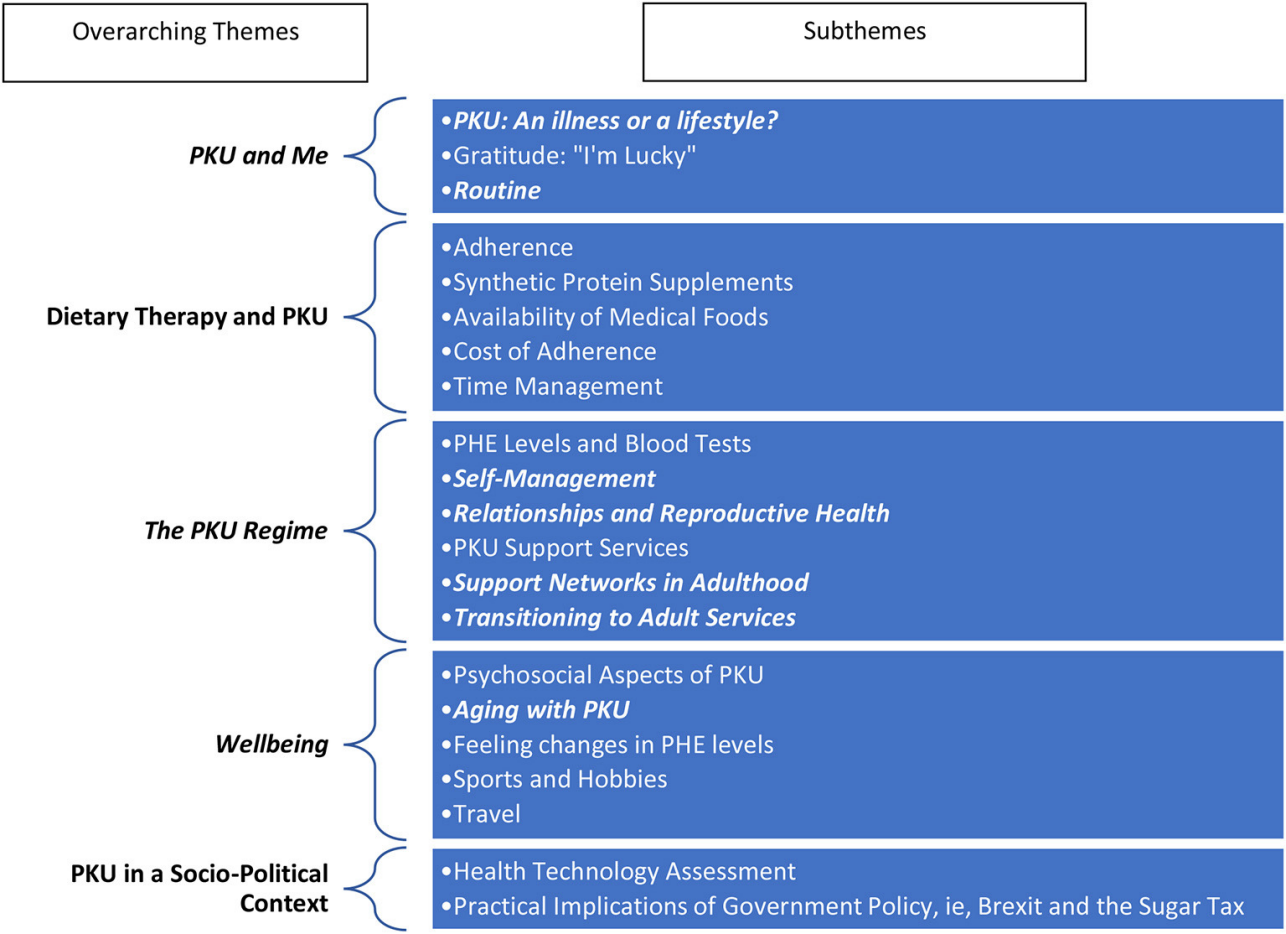
Overall study findings, overarching themes and subthemes^*^.

* Themes in *italics and bold text* represent the themes discussed in this paper.

### Ethics and reflexivity

The research was conducted in accordance with the University's research ethics policy and the core bioethical principles of autonomy, beneficence, non-maleficence and justice (Beauchamp and Childress, 2013) were upheld throughout. Ethical approval was obtained from the University research ethics committee. Formal gatekeeper approval from the PKUAI was sought from official board members independent of the current Chairperson, who was the principal supervisor on this project. Additionally, the co-supervisor chaired the University research ethics committee. For transparency, the co-supervisor removed herself from committee decisions relating to this project in order to avoid influence. The combined supervisor roles proved to be simultaneously advantageous and challenging, as it required a high degree of reflexivity and bracketing to be practiced within the group.

The researcher informed all research participants of the principal supervisory role within the study for the purpose of research transparency and to facilitate fully informed decision making. All research participants were made aware of their right to withdraw from the study at any time. Each participant signed the consent form and consented to the use of an audio recording device for the duration of the interview. Each participant was advised that the research findings may be published on completion of the research study. All names of individuals or healthcare organizations have been removed from verbatim transcripts in line with the Data Protection Act (2018). Cognizant of the small nature of the PKU community, transcripts were also screened for any potential secondary identifiers. Additionally, a strong emphasis was placed on the avoidance of secondary identifiers when discussing research participants within the supervision process, particularly during data analysis. This approach was guided by the co-supervisor who has a clear understanding of research ethics and no affiliation with the PKU community.

## Findings

Within qualitative research, the recommended number of participants varies but is estimated to be between 10 and 12 participants, or, until data saturation is reached (Marshall and Rossman, 2016). Moser and Korstjens (2018) suggest data saturation can determine sample size and, therefore, differs with each study. The current research study reached data saturation having recruited and interviewed (n = 11) participants.

The original study findings highlighted barriers and facilitators to living with PKU at various points throughout the lifespan of research participants. These included adherence to DT, transitioning from child to adult services and the experiences and complications of moving through adulthood with PKU, including reproductive health. The current article will explore the findings which appear to be least represented within the existing literature: living with PKU; transitioning from child to adult specialist services; routine and being self-sufficient; support networks in adulthood; aging with PKU.

### Living with PKU

There was unity among the vast majority of research participants, who considered PKU to be a part of themselves or part of their lifestyle. In all cases, research participants agreed that PKU was a cause of increased difficulty in life, however, some expressed relief that they did not have, what they considered, more serious health conditions. The following excerpts represent these views:

“*It just is what it is. It's like your hair is brown or whatever, you know what I mean. It's part of me. I don't necessarily think of it as an illness but it's part of me.” P3*

“*People call it a disease and people call it an illness, I don't. I call it a metabolic disorder; I think of it just as a diet for life. I don't think of myself as having a disease. Technically it might be but I don't have that opinion. I don't see as much of a disability either in terms of the scale of what you can be born with, it doesn't even begin to register really.” P9*

Such views may be supported by a diagnosis of PKU at birth, for which participants demonstrated a sense of gratitude as they reflect on neonatal testing availability in Ireland:

“*I'm lucky, I have been lucky.” P1*

“*I've never known anything different. I was diagnosed at birth, which is, I deem it to be a blessing.” P6*

There was an evident tension in identifying PKU as an illness or a lifestyle. The following quote embodies the conflicting views and the complicated relationship with PKU as experienced by research participants:

“*Sometimes I think it depends on how you're feeling at the time. If you're finding it hard to control it [PKU] and you're feeling run down and your [PHE] levels are high and you're not feeling well then you consider it an illness. And then other times when everything's okay, and you've control and you're feeling good, then you're not really considering it an illness. So, I think it depends on how its impacting you at the time.” P11*

All research participants related this concept to their own experiences of living with PKU and the possible barriers and facilitators to living well with PKU. Even though research participants differed in their views, all identified difficulties associated with PKU that interrupted their everyday lives.

“*There's illnesses that are far worse that I could have, that are debilitating physically and stuff. But it does affect your quality of life a lot and your ability to do certain things.” P5*

“*You could call it an illness, you can call it a condition, you can call it whatever you want. It's something that has to be maintained. If you are not looking after your PKU, you're not looking after your health.” P1*

“*I see it as an illness, a disease, it's something that affects your brain, your mental health.” P10*

When exploring what it was like to be a man or a woman living with PKU, the experience of reproductive health was highlighted as the most distinguishing factor between genders:

“*I suppose for females with PKU, the added difference is the whole area of pregnancy with PKU... It's an extra worry. It's an extra fear in the case of an unplanned pregnancy.” P11*

Each participant was asked if having a diagnosis of PKU had influenced their decision to have children. Again, the responses were startlingly contrasting:

“*I haven't been put off having kids or anything from it [PKU].” P5*

“*Yeah it has affected [my decision] it's something I'd really have to think long and hard about, it [PKU] is something that's kind of put me off nearly having kids.” P11*

Similarly, the knowledge and information displayed by participants regarding their reproductive health varied among the genders. Whilst female participants were very aware of maternal PKU and the associated risks, one male participant was completely unaware of the mode of inheritance for PKU:

“*I definitely wouldn't [have children]. I think it would be hard if you had children with PKU. I just remember reading something that like PKU has an effect on offspring. If the woman giving birth has high [PHE] levels. But I've never seen anything that men would [sic], have an effect on the child. I don't know how it [mode of inheritance] works.” P8*

The most prominent concern among female research participants was fear of causing harm to the baby in utero, due to their own negligence of DT. Female participants that did not have children reported that they would like to, however, they felt that a fear of pregnancy had been engrained into them from a young age. One participant noted:

“*We were in [healthcare services] and the [staff] were like, ‘So basically, if you don't manage your pregnancy, but even if you do come straight to us… But here's a leaflet of all the things that can go wrong'. And I just wept, I broke down crying... I could never ever bring a child into the world if I thought that I did something wrong to them. And it was just very hard for me psychologically, because I've always wanted kids.” P1*

### Transitional period

Two research participants were undergoing the process of transitioning from child to adult specialist health services at the time of data collection:

“*I am in limbo at the moment. I don't have anywhere [service provision]. [I'm] 22 and I'm still in the kids hospital... I don't know where to go.” P1*

The majority of research participants reported recently returning to adult support services having disengaged from pediatric support services a number of years prior. The re-engagement process was reported as tedious and long. The term “*left in limbo”* had been used repeatedly with reports of waiting up to 2 years for an initial appointment with support services:

“*That was a disaster for me, the transfer... I was in limbo for about two and a half years.” P4*

Despite the lengthy and tedious transitional period, the majority of research participants reported receiving invaluable support and assistance from the adult healthcare services, once the transition process was complete:

“*They are more collaborative.” P2*

“*They start talking to you more about the whole science and implications of not being on diet properly and explaining things.” P5*

Research participants who had gone off diet claimed healthcare staff largely contributed to their return to diet by providing education, dietary and psychological support:

“*They've told me that I can ring at any time I want to talk, or if I'm finding it tough... They have had a lot to do with me getting the diet back on track and making it more manageable.” P4*

“*I find that the dieticians are very good in terms of helping you.... like when I was trying to manage my diet they would go through [my] activities and try and work out how much of the formula [I] should be taking, so that [I'm] getting the correct protein intake. And also, they worked with me a lot to try and balance my diet so that it was more healthy [sic].” P5*

However, as with all services, opinions vary. Some issues raised included distance to clinic, delay in receiving blood PHE results, the repetitive nature of consultations, reduced contact hours and the provision of a psychological support worker that was only available part time, at the time of this study. Yet, overall, the adult services were attributed with satisfactory support, particularly with regards to managing blood PHE levels, providing education, and making the return to the low protein DT more manageable.

### Routine

Consistent daily routine was attributed to successful metabolic management and reduced levels of stress and anxiety. The majority of research participants reported having the same breakfast every morning as part of a “*budgeting system*,” facilitating the negotiation of daily protein allowance:

“*I tend to have the same breakfast day in and day out because it means that I can monitor what I'm actually ingesting...I tend to measure it out. So, I can budget, it's a budgeting system!” P6*

Research participants who were very physically active were more likely to rely on a daily routine for metabolic management. The routine did not solely consist of preparing meals in advance but specific timing of synthetic protein supplements to compliment endurance or recovery in their preferred activity:

“*Before the [football] game I drink a half one [protein supplement] because it's a bit heavy going into games and stuff... After the game I drink the full thing and I'd [sic] have it immediately, as soon as possible.” P8*

The synthetic protein supplement was identified by research participants as the foundation block of living well with PKU. Despite recognizing the advantages of being compliant with the synthetic protein supplement, research participants identified physical and social implications of taking it:

“*I remember being very young and thinking that I'd never kiss someone because of my drinks [due to the odour].” P1*

“*I got to the situation where I go off to the loo to have my drink.” P7*

The majority of research participants confirmed that the palatability and transportability of supplements had improved significantly. However, a number of side effects were described including gastrointestinal issues, nausea and dental erosion. Four research participants reported buying low protein food products online in an attempt to vary the low protein diet, however, this came at a high cost:

“*I bought some [Brand Name] stuff that was like yogurt drink, that was quite nice, and the breakfast bars and stuff. But it was about* €*45 for just the two of them. And it was only six breakfast bars and one tub [of yogurt].” P3*

### Becoming self-sufficient/self-management

Managing the PKU regime requires a large degree of self-management. Research participants often described moving between adherence, non-adherence and partial adherence to DT at different points throughout their lives:

“*The toughest time, for a lot of people, seems to be around like 18 to 25 ish... Like, honestly I was off the diet a lot around times there.” P5*

“*I'd never come off diet, I've always stuck to diet. But I think I've been over-exchanging… even when I thought I was having good days, [I was] still over exchanging.” P4*

Research participants described what they believed to be adherence to DT, but described limited usage of low protein food products in favor of natural protein sources:

“*I'm trying to eat as natural as possible. To do that requires me, really, to break my diet.” P3*

Additionally, research participants reported feelings of cultural exclusion during shared meals with family members or peers, as a direct result of DT and social exclusion associated with eating out:

“*It's not that I eat out a lot, it's just that that's the most difficult thing.” P1*

“*[If] I was going for meals with my friends at the weekend, or going out drinking, because you're just like, ‘oh doing this with the guys', and you just feel a bit isolated and not part of what's going on. It's not a peer pressure but it's like a self-imposed sort of pressure that you don't want to look or feel different.” P5*

### Support networks in adulthood

The majority of research participants attended PKUAI meetings and appeared to have a better insight into the broader context of PKU than their counterparts who did not. They were also more informed regarding new developments in treatment and additional support services. The PKUAI was attributed with campaigning to attain better care and services for people with PKU living in Ireland and encouraging advocacy, self-management and peer support:

“*Most of the information I get actually is from the PKU Association.” P5*

One unanticipated finding was that all research participants reported seeking support *via* social media platforms, with the majority reportedly getting their information on PKU related issues from online forums. This instilled feelings of community and inclusion:

“*I hadn't spoken to anyone or seen anyone talk about it until I saw that blog.” P1*

However, research participants highlighted exposure to negative influences when engaging with some online platforms, such as PKU support groups, arguing that such engagement promotes a negative outlook on living with PKU:

“*I'm a member of two groups on Facebook… I see people on that who believe their PKU is the cause of all the ills in the world. I don't visit the online groups that much at the moment, too much negativity there.” P9*

This perceived negativity apparently encouraged participation in the current research study as participants wished to promote the positive aspects of living with PKU.

### Aging, what happens next?

Concerns regarding the effects of aging on an adult with PKU was overwhelmingly apparent for research participants who muted concerns regarding the possible negative effects of PKU in older age. Although PKU was often considered a lifestyle by research participants, it was recognized as something that may need more care and attention as participants get progressively older. The following quote is from a research participant discussing the psychological worry and stress of aging with PKU:

“*As I get older now, that is something I worry about long term, is how I'm going to be when I'm like, 60's. Like eventually, am I going to wear down parts of my brain even though I'm not that far over my limits. That does come with a lot of stress as well thinking long term about things. Because there's not a lot of research about that, about how it affects people in old age.” P5*

A number of older adult research participants are regarded as some of the first to be diagnosed with PKU since the introduction of neonatal testing in Ireland in 1966. With the initiation of DT and neonatal testing, these adults are some of the first in Ireland to reach this stage of their lives without apparent developmental or cognitive delays. This realization, the awareness that they are the oldest adults living well with PKU in Ireland, and the lack of research available exploring the aging PKU population, presented as a primary concern for these participants:

“*I'm one of the earlier ones, there are older than me, but there's not really much research done as to what happens later on in life to PKUers.” P2*

Concern caused by a lack of research in the area of aging with PKU was tangible:

“*At my stage in life, I just feel like there's not really much research done into the older end of the spectrum. So, I'm kind of blocking that out of my mind...What effect does that have when I'm 60 or 70? Is [my son] gonna [sic] have to mind my dribbling mouth, wipe me up, and, you know? I don't know. It's a mystery because nobody does the research.” P2*

A female participant alluded to another aspect of aging which is poorly understood in the context of PKU:

“*Menopause is around the corner for me, I don't know what's going to happen then. There's no research, there's no backup.” P2*

Research participants who had disengaged from healthcare services were encouraged to re-engage over concerns for their neuro-cognitive and physiological functioning, or a perceived decline thereof. There were significant concerns by some who described noticing a decline in short term memory functions:

“*I do think [PHE levels] do damage to your short-term memories…I do think I did some damage.” P9*

The excerpt below is from an adult who reports these concerns encouraged re-engagement with healthcare support services:

“*More recently, I've become concerned about brain function, brain health into older age and especially the knock-on effect [of] all this extra protein hacking your brain or getting at your brain because the blood brain barrier. So, I've gone back on it [the diet] and got my [PHE] blood levels back down.” P9*

However, similar concerns for the future were expressed by younger research participants, aged from late twenties onwards:

“*I've never heard any, or had any discussion about, like, does it put you at risk [of] brain conditions like Dementia or Alzheimer's and things. I think at the last meeting, someone who was at the ESPKU, I think he said there were some researchers said there wasn't any link. That sort of puts your mind at ease about things like that, but you always still worry.” P5*

### Hope

It was reported by research participants that research within the area of aging and PKU was limited, which had encouraged their participation in the current research study. Surprisingly, fear of aging was associated with hope for the future. Participant's were aware of developments in treatment for PKU, this instilled a feeling of hope:

“*I'll be optimistic that there'll be great developments in the near future.” P6*

Within the discussion of aging, it was interesting to note how often research participants mentioned new parents of children with PKU and how they themselves felt they could provide solace or comfort:

“*I offer my availability if it was, [to be a] link for the new parents because I know new parents can be quite anxious about what is PKU... Because now we've lived with it and we've got to this stage in our life. We are fully functioning adults out in the big wide world and walking down the street. Everybody looks the same, there's no difference to almost anybody else. That would be nice… to take the fear factor away from new parents... Its more to say, if you have PKU, you can have a job. You can have kids; you can do all the normal stuff people out there do. It's not all doom and gloom.” P7*

Aside from reassuring parents of children with PKU, another recurrent theme was that research participants were determined to let people know PKU did not control their lives and they lead lives much the same as the general population. A rather significant result to emerge from the data was the extent to which adults with PKU claimed to want to participant in the research study in order to get this point across:

“*What I'd like to tell people is that listen, it's fine. It doesn't change much. It changes your diet and your lifestyle a small bit. But it still doesn't define the person your kids going to be. It doesn't define what they have to do, or what they can't do. They can still do anything and everything they want, they can travel the world if they want, I did it like, you know. And other PKU people have done it.” P4*

“*I think academically and career wise, I haven't found that the diet has held me back in life so far.” P5*

Other research participants reported taking part in the current research study as they wished to see if they could relate with how other adults perceived their lives with PKU:

“*It's nice to get the opinion of adults that have gone through the system. I'd have to make it better for the people coming after, you know, and later on in life...In that sense I'd like to, you know, start giving back.” P4*

## Discussion

### Living with PKU

Phenylketonuria, by its definition, is a hereditary metabolic disease (World Health Organisation, 2019). However, Seidlein and Salloch (2019) suggest the concept of illness and disease are often subjective and dependent on a person's understanding and experience of health. Furthermore, Borghi et al. (2020) identified PKU as a prominent element in the identity of AwPKU. Similarly, the majority of research participants considered PKU to be a part of themselves or part of their lifestyle. It was identified within the findings that the perception of PKU being an illness often correlated to how well adults could maintain metabolic control. PKU was more likely to be considered an illness if adults were struggling with the PKU regime, therefore, PKU was more likely to be perceived as negatively influencing the lived experience. This is reflective of the wider health and illness discourse within the literature. The medical model is the predominant approach to illness management, however, Branitska et al. (2019) highlight disadvantages associated with this approach. To refer to an individual with a diagnosis as being unwell is an objective assumption without any regard to the subjective experience (Nordenfelt, 2007). How an individual with a chronic condition experiences living with that condition can be influenced by ‘illness intrusiveness' (Kalfoss et al., 2019). The concept of illness intrusiveness is a psychological construct influenced by one's perception of the degree in which illness disrupts lifestyle. Research suggests that a person's insight into coping with a chronic illness largely influenced perceptions of illness intrusiveness in self-evaluations (Shawaryn et al., 2002; Lebrun et al., 2016). Similarly, a correlation has been identified between illness intrusiveness and quality of life as experienced by individuals with chronic conditions (Kalfoss et al., 2019). Moreover, Khoso et al. (2016) make clear distinctions between health behavior and illness behavior. Health behaviors are the daily routine behaviors and lifestyle of an individual that maintain current health status and determine health threats. In contrast, illness behavior develops with feelings of unwellness and actively seeking a cure. Additionally, Roberts et al. (2020) has estimated that stigma associated with illness leads to difficulty communicating about the condition and an altered perception of social inclusion.

Research participants of the current study compared PKU to what they deemed to be ‘*more serious conditions'* and expressing gratitude for receiving a diagnosis at birth. The idea of having grown up with PKU may have influenced these perceptions. These findings are consistent with research wherein participants did not consider PKU to be an illness, having received a diagnosis at birth (Vegni et al., 2010; Diesen, 2016; Cazzorla et al., 2018). The sense of gratitude expressed by research participants resonates with the findings of Diesen (2016) who suggests that a sense of gratitude could be testament to significant coping strategies and facilitates a holistic view of the lived experience of AwPKU.

### Reproductive health

As healthcare knowledge expands, Beecher et al. (2019) argues the delivery of healthcare and health education has become more complex. Govender and Penn-Kekana (2008) identify patient gender as a primary influence of interactions with healthcare providers and mirrors the wider sociological constructs. Similarly, WHO have declared worldwide governmental strategies for providing sexual health education are predominantly aimed at young women (World Health Organisation, 2016), as was evidenced by research participants. According to Murphy (2015) treated PKU does not affect fertility, however, unmanaged PKU can lead difficulties with the pregnancy and an increased risk to the fetus. The risks associated with untreated maternal PKU include microcephaly, facial dysmorphia, stunted growth and congenital heart disease, associated with elevated PHE levels in the mother (Levy, 2003; Van Wegberg et al., 2017; Yildiz and Sivri, 2019). As Munyame et al. (2018) explains, a child born with PKU can be treated through DT, however, damage caused to a child or fetus within the womb, due to mothers elevated blood PHE levels, is irreversible. In order to combat these risks, a number of literary sources, including the *The Complete European Guidelines on Phenylketonuria*, recommend dramatically reduced therapeutic PHE blood levels for the mother and stricter adherence to DT to commence preconception (Van Wegberg et al., 2017; Yildiz and Sivri, 2019). The most prominent concern among female research participants was fear of causing harm to a baby in utero due to their own negligence of DT. This concern was also reported by Bosch et al. (2015) and Ford et al. (2018b). However, Ford et al. (2018b) found the rigorous pre-conception and pregnancy routine to result in heightened levels of stress and anxiety for the mothers, leading to a perceived reduction in the quality of life experienced by women with PKU. Additionally, Ford et al. (2018b) found that women with PKU were more likely to report non-adherence to the low protein diet post pregnancy, due to trying to manage the demands of a new-born baby and DT. Despite Munyame et al. (2018) declaring that the management and prevention of maternal PKU is sufficient, there is evidence within the current study that fear of pregnancy is prominent in the lives of women with PKU. Additionally, these findings strongly support those of Ford et al. (2018b) who assessed the reproductive experience of women with PKU.

According to the research findings, male research participants did not experience the same levels of emotional distress described by female participants or within the literature, when discussing reproductive health. It was difficult to ascertain if these findings mirrored the attitudes of other adult men with PKU as the majority of pregnancy and PKU research pertains to women and risks associated with maternal PKU. According to early studies by Fisch et al. (1991) and Levy et al. (1991), fetal abnormalities have not been associated with paternal PKU. However, research surrounding paternal PKU appears to be very limited with no recent development being reported. Moreover, no research could be found on the male experience of paternal PKU. Considering the vastly conflicting experiences of men and women with PKU discussing reproductive health and relationships, a possible explanation may be that males with PKU do not receive the same levels of sexual health education and genetic counseling from their metabolic teams. McGowan et al. (2020) has determined a need to improve preconception awareness for both young male and female adults by refocusing the timing, platforms and educational tools used to deliver preconception health awareness. A primary issue emerging from the current research findings are the possible long-lasting effects of sexual health education delivery by healthcare teams and the apparent gender inequality. As the findings associated with education received from healthcare services are of a reflective nature and possibly no longer as influential, it is an important issue for future research and there may still be some unanswered questions surrounding the experiences of fertility, reproductive health and relationships for adults with PKU. To develop a more in depth understanding, future studies carried out should assess how sexual health education and genetic counseling are presently delivered. There is also an identified need for sexual health education and research in the context of males with PKU.

### Routine

Consistent daily routine was attributed to successful metabolic management and reduced levels of stress and anxiety by research participants. Similarly, Mutze et al. (2016) found many AwPKU find it increasingly difficult to remain adherent to DT if their routine has been interrupted. Nonetheless, the importance of daily routine is seldom referred to within the literature pertaining to PKU management. Knowing what to eat but trying to maintain an ‘optimal eating pattern' has been described as a major challenge for people with long term conditions being treated by any form of DT (Forouhi et al., 2018). The wider literature suggests the establishment of daily routine has been shown to reduce stress levels and depressive symptoms (O'Conor et al., 2019; Widnall et al., 2020), improve dietary intake (Singh et al., 2020) enhance sleep (O'Conor et al., 2019) and positive wellbeing (Heintzelman and King, 2019) for all individuals irrespective of illness. O'Conor et al. (2019) suggests the presence of daily routine may positively influence self-management and improve health related outcomes for individuals with life-long conditions.

### Self-management and the transitioning process

Effective self-management skills have been widely recommended for people with life-long conditions (Hughes et al., 2020). Self-management refers to one's own ability to regulate thoughts, feelings and behaviors by utilizing skills such as planning, organization, goal setting and stress management (Pincha and Ayra, 2013; Mitchell et al., 2019). AwPKU are encouraged to fully self-manage all aspects of the PKU regime by the age of eighteen (Trahms, 2008). Moreover, successful self-management is co-ordinated by the individual with support from family members and healthcare providers (Health Service Executive, 2017). Trappenburg et al. (2013) are of the opinion that self-management support from healthcare providers is essential in order to attain health as it is defined by Huber et al. (2011) and World Health Organisation (2020).

Transitioning is a term used in healthcare to describe the planned process of transfer for a person with a lifelong condition from child to adult specialist services. It has been shown that the transitioning period often leads to disengagement with services (Mutze et al., 2016). Furthermore, specialized metabolic centres have reported decreased adherence to dietary management and an increase in loss of clients to follow up care as patients grow older (Hofmann and Barker, 2017). Eating out with peers was considered a primary contributing factor to reduced or non adherence to DT by research participants. These findings are consistent with research by Alptekin et al. (2018) and Ford et al. (2018a) whom suggest links between the social consumption of food and anxiety. Moreover, Sattoe et al. (2014) argue the transition to adult services coupled with increasing responsibility can have a negative impact on the quality of life experienced by people with long term conditions. There is substantial literature which suggests poor transitioning into adult services for young adults with life-long conditions is universal (Campbell et al., 2016; Kerr et al., 2018; Robertson, 2019; Colver et al., 2020). The term “*left in limbo*” had been used repeatedly by research participants with reports of waiting up to 2 years for an initial appointment with support services. Moreover, Kerr et al. (2018) found poorly planned transitions for people with life-long conditions result in negative social and emotional outcomes and poor or non-adherence to treatment plans. Traditionally, a low success rate has been associated with adults attempting to reinstate DT (Bik-Multanowski et al., 2008). However, research participants who had previously discontinued DT claimed healthcare staff largely contributed to their return to the low protein diet by providing education, dietary and psychological support. These results are in accord with recent studies indicating engagement with follow up care leads to better metabolic and clinical outcomes for AwPKU (Mutze et al., 2016; Cazzorla et al., 2018). Furthermore, good family relationships coupled with teaching “good behaviors” are shown to encourage adherence to DT in young people with PKU (Witalis et al., 2017). Aside from healthcare staff and the PKUAI, research participants reported getting their information on PKU related issues and seeking online peer support from social media platforms and online forums. Peer support provides an honest expression of thoughts and feelings, a source of empathy (Legg et al., 2019) and shared lived experience (Fortuna et al., 2019). It is possible that peer support *via* social media interaction is preferable as it takes away the medical professional and clinical aspect of a diagnosis of PKU. However, it also facilitates exposure to negative experiences and mentality of others (Legg et al., 2019), as highlighted by research participants. Further insight in the context of online peer support would help to further explore this relationship.

### Aging and hope

Mitchell (2019) suggests the presence of illness makes one aware of their own mortality. This was apparent for research participants where concerns regarding the possible negative effects of PKU in older age were muted. Despite their contribution to the current research study and the recommendations of DT as a diet for life, there is currently limited research investigating the long term impact of DT on neurological or physiological functioning, therefore, it remains fully supported as a diet for life (Van Spronsen et al., 2017; Pilotto et al., 2019). However, having conducted a review of the available literature, Vardy et al. (2020) question any associated effects of DT at different stages throughout the life span and whether DT can maintain health in older AwPKU.

Brain pathology has become the focus of PKU research in hopes of predicting future outcomes. Although EU guidelines (Van Wegberg et al., 2017) declare current neuroimaging techniques are not adequate in monitoring clinical outcomes for adults with PKU, they recommend routine neuro-cognitive evaluations should be carried out when a person with PKU reaches the ages of 12 and 18 years to determine baseline neuro-cognitive functioning. Burlina et al. (2019) has shown neuroimaging of the PKU brain in early treated AwPKU have shown abnormalities in brain matter physiology when compared to controls. Similarly, Pilotto et al. (2019) has identified negative correlations for neurotransmitter atrophy and PHE levels in early treated adults with PKU suggesting an acceleration of brain damage due to aging. According to Rudzińska et al. (2020), a direct relationship has been established between gene mutations and age-related conditions, however, the effects of aging remain unclear for many pathological conditions due to the complexity of the aging process.

According to Balen and Merluzzi (2021) hope is transferable across everyday life and within the context of living with rare or long-term conditions. Hope is regarded as an important component in the treatment of chronic conditions, having been associated with improved quality of life, personal coping strategies and resilience in people with serious long-term conditions (Solano et al., 2016; Pais Ribeiro and Pedro, 2022). Similarly, uncertainty can be associated with vulnerability, fear and anxiety (Hillen et al., 2017). At present, there is a paucity of literature investigating the effects of PKU in older age or the experiences of an aging PKU population. Albeit, Kruse (2020) argues that human existence is characterized by vulnerability and potentiality of aging outcomes. Gruhn et al. (2015) report older adults with complicated medical conditions have a limited future time perspective when compared to younger counterparts or ‘healthy' older adults. Additionally, time perspective is associated with negative emotional regulation in despite of successful health management (Gruhn et al., 2015). Moreover, O'Brien et al. (2017) suggest individuals' attitude to aging can influence health related and psychological outcomes. It could be suggested that the chronological age of research participants directly influences their perspective of future time and their emotional regulation in relation to aging with PKU. Due to lack of research in the area, Pilotto et al. (2019) acknowledges adulthood and aging with PKU can be a vulnerable experience. In order to address concerns and questions relating to aging with PKU, Vardy et al. (2020) urge the creation and maintenance of PKU registries and longitudinal studies in the area of aging with PKU be carried out.

## Conclusion and recommendations

It was evident from the findings that even though research participants regarded PKU as being part of their identity, living well with PKU is complex. The current paper offers new insight into the vulnerability attached to the experience of living and aging with PKU. A number of recommendations have been made in key areas for future practice, policy and research:

With regards to the transitional process, research participants reported the period between late teens and early twenties was indicative of non-adherence with DT, therefore, future research should focus on determining what influences this behavior and establish how to provide sufficient support to this age group. Similarly, comparative studies exploring this age group and the transitional period for other rare conditions may help to inform all stakeholders. Moreover, a review if the Irish National Clinical Programme for Rare Disease (Department of Health, 2014) for specialist healthcare service provision is advisable. Additionally, a male nurse/healthcare worker and genetic counselor/genetic counseling service should be made available and easily accessible for AwPKU. Similarly, research with a view to elicit the experience of fatherhood, or consideration prior to conception for men with PKU, may help to inform healthcare providers and the general PKU population of Ireland. The provision of full-time psychological support is also desirable. Furthermore, an exploration of how sexual health education and genetic counseling are presently delivered could help to inform future educational practices. A public and patient involvement (PPI) approach should be considered in the co design of educational materials to enhance the experience of AwPKU. It is hoped these recommendations would enhance the patient experience.

Research on the benefits of daily routine for people with PKU may be beneficial for future development of self-management education. Additionally, future research should determine if a relationship exists between increased physical activity and lower perceptions of illness intrusiveness. Moreover, extensive longitudinal mixed method research with the use of MRI brain imagery, PHE level measurement and QoL measurement tools could be used to develop targeted interventions aimed at preserving brain pathophysiology in the older adult with PKU.

Finally, research wherein a greater focus is on the use of social media platforms and the positive and negative aspects of unsolicited peer support could produce interesting findings.

## Strengths and limitations

The primary limitation of this study was the small number of participants. Furthermore, the majority of research participants were male, therefore, the experience and concerns of women with PKU may be underrepresented. This includes AwPKU who have limited or no internet access. However, as is more typical of qualitative research, the use of personal narrative recognizing cultural norms may resonate with the wider PKU population of Ireland. Notwithstanding the relatively limited sample, this work offers valuable insights into the lived experience of PKU within an Irish context.

## Data availability statement

The raw data supporting the conclusions of this article will be made available by the authors, without undue reservation.

## Ethics statement

Ethical Approval was obtained from the University Research Ethics Committee prior to commencement of project. Formal gatekeeper approval was obtained from the PKU Association of Ireland prior to recruitment. The patients/participants provided their written informed consent to participate in this study.

## Author contributions

BSG and A-MG contributed to conception of the research study, acquiring funding and support, and played a significant supervisory role. MEO'S played a leading role in carrying out the research and collection of data. AMCD assisted in the development of the article for publication. All authors contributed to the interpretation of data and contributed to the final manuscript.

## Funding

This research was funded by the Institute of Technology Tralee (now Munster Technological University) under the President's Postgraduate Research Scholarship Programme. Funding for publication was granted by the Munster Technological University.

## Conflict of interest

Author BSG is the current Chairperson of the PKU Association of Ireland and was not Chairperson at the time of the research but was a spokesperson for the PKUAI and did not take an active role in attaining gatekeeper approval or participant recruitment. The involvement of MEO'S and AMG, not connected with the PKU community, and an appreciation of reflexivity within the research, and the supervision process, both acted as factors to minimize any concerns in this regard (Hofmann and Barker, 2017). The remaining author declares that the research was conducted in the absence of any commercial or financial relationships that could be construed as a potential conflict of interest.

## Publisher's note

All claims expressed in this article are solely those of the authors and do not necessarily represent those of their affiliated organizations, or those of the publisher, the editors and the reviewers. Any product that may be evaluated in this article, or claim that may be made by its manufacturer, is not guaranteed or endorsed by the publisher.
